# Bacterial Involvement in Oral Squamous Cell Carcinoma and Potentially Malignant Oral Disorders

**DOI:** 10.1111/odi.70115

**Published:** 2025-10-09

**Authors:** Atsumu Koketsu, Satoshi Fukase, Toru Tamahara, Tatsuru Saito, Akiko Ito, Yutaro Higashi, Tomonari Kajita, Tsuyoshi Kurobane, Masaaki Miyakoshi, Masahiro Iikubo, Kazuki Kumada, Bin Li, Muneaki Shimada, Ritsuko Shimizu, Tetsu Takahashi, Kensuke Yamauchi, Tsuyoshi Sugiura

**Affiliations:** ^1^ Division of Oral and Maxillofacial Oncology and Surgical Sciences, Department of Disease Management Dentistry Tohoku University Graduate School of Dentistry Sendai Miyagi Japan; ^2^ Tohoku Medical Megabank Organization Tohoku University Sendai Miyagi Japan; ^3^ Division of Dental Informatics and Radiology Tohoku University Graduate School of Dentistry Sendai Miyagi Japan; ^4^ Advanced Research Center for Innovations in Next‐Generation Medicine Tohoku University Sendai Miyagi Japan; ^5^ Division of Oral and Maxillofacial Reconstructive Surgery, Department of Disease Management Dentistry Tohoku University Graduate School of Dentistry Sendai Miyagi Japan

**Keywords:** leukoplakia, microbiota, mouth neoplasms, oral cancer, oral lichen planus (OLP), oral potentially malignant diseases (OPMDs), squamous cell carcinoma of head and neck

## Abstract

**Objective:**

To clarify the relationship between oral squamous cell carcinoma (OSCC), potentially malignant oral disease (OPMD), and bacterial flora using metagenomic analysis.

**Methods:**

This cross‐sectional observational study included 50 patients in the control group and 77 patients with OPMDs, 41 with early OSCCs, and 20 with advanced OSCCs. Patient saliva samples were subjected to high‐throughput sequencing of 16S rRNA gene amplicons to evaluate the composition and diversity of the oral microbiome.

**Results:**

No significant differences were observed in patient backgrounds, other than sex. Patients with advanced OSCCs had greater oral bacterial diversity than those with early OSCC or OPMD. The advanced OSCC group formed a distinct cluster separate from the other groups. Sixteen and 275 species were identified at the phylum and genus levels, respectively. Compared with the control group, *Actinomycetia* and *Streptococcus* were significantly elevated in the early OSCC and OPMD groups. *Peptostreptococcus* and *Fusobacterium* were significantly higher in the advanced OSCC group than in the control, OPMD, and early OSCC groups.

**Conclusions:**

The composition and diversity of oral microbiota may be associated with OPMD development and progression to OSCC. Consequently, the salivary microbiome may serve as a biomarker for oral cancer and help predict cancer progression.

## 1 Introduction

The risk factors associated with squamous cell carcinoma include tobacco, alcohol, and human papillomavirus (HPV) and epithelial papillomavirus infections (Johnson et al. 2020; WHO Classification of Tumours Editorial Board 2022). Among the various squamous cell carcinomas, oral squamous cell carcinoma (OSCC) may arise from a subset of oral potentially malignant oral diseases (OPMDs), which are clinical conditions associated with an increased risk of malignancy. Leukoplakia is the most common OPMD (World Health Organization 2024; WHO Classification of Tumours Editorial Board 2022). Leukoplakia is defined as “a predominantly white plaque of questionable risk having excluded (other) known diseases or disorders that carry no increased risk for cancer,” as proposed by Warnakulasuriya et al. (2007, 2021). It is a clinical diagnosis of exclusion and cannot be attributed to specific causes. Histopathologically, leukoplakia may or may not present with epithelial dysplasia. Although the risk of malignant transformation associated with epithelial dysplasia in oral leukoplakia depends on the severity of dysplasia, it remains probabilistic rather than certain. Meta‐analyses estimate that the cumulative malignant transformation rate of leukoplakia is approximately 10%, or 1%–1.5% per year. Moderate to severe dysplasia carries ~2.4‐fold higher risk than mild dysplasia, whereas high‐grade dysplasia carries ~5‐fold higher risk than low‐grade dysplasia (Carlson et al. 2023; Warnakulasuriya 2018; WHO Classification of Tumours Editorial Board 2022). The innate immune system in the oral environment protects against the invasion and establishment of exotic pathogenic microorganisms, including bacteria, viruses, and fungi (Arthur et al. 2021; Bouvard et al. 2009; Chu et al. 2017; Kageyama et al. 2019; Kelly et al. 1993; Kouketsu et al. 2019, 2023; Shoemark and Allen 2015; Warren and Marshall 1983).

The microbiome is among the most important factors contributing to the regulation of host health, and numerous studies have revealed the mechanisms by which microorganisms affect its general condition (Emoto et al. 2016; Johnsen et al. 2018; Qin et al. 2012; Yachida et al. 2019; Yamashiro et al. 2021). The oral microflora is the second most diverse after the intestinal microflora, with more than 500 species of bacteria inhabiting the oral cavity (Wang et al. 2019). In contrast, the diversity of bacterial species is the lowest in mucosal flora, followed by saliva and dental plaque (Keijser et al. 2008). Studies have also identified the involvement of specific microorganisms in gastrointestinal tract carcinogenesis. For example, in addition to *Helicobacter pylori* in gastric cancer, *Fusobacterium nucleatum* , which causes periodontal disease, is also characteristically present in the stools of patients with colorectal cancer (Rubinstein et al. 2013). Moreover, whole‐genome analysis of oral microflora (bacterial flora) has also made dramatic progress.

Several studies have investigated the relationship between the oral microbiota and systemic diseases, with an increasing focus on their role in oral carcinogenesis. For example, Yang et al. (2017) suggested that saliva from patients with cancer was enriched with specific bacterial taxa. Subsequently, recent studies reported differences in the salivary microbiome among healthy individuals, patients with epithelial dysplasia, and patients with OSCC (Radaic et al. 2024; Galvin et al. 2025). Despite accumulating evidence linking the oral microbiome with OPMDs and OSCC, only a few metagenomic analyses have examined microbial dynamics across distinct disease stages. Therefore, this study aimed to clarify the relationship between the oral microbiota and development of OPMDs using saliva samples from patients with oral cancer and metagenomic analysis. The present study addresses the research gap by demonstrating associations between salivary microbiota and OPMDs/OSCC and highlighting their potential as predictive biomarkers in oral carcinogenesis.

## 2 Materials and Methods

### 2.1 Patients

The study protocol was approved by the Ethics Committee of Tohoku University Graduate School of Dentistry (No. 25091). The study design and objectives were explained to the patients, and written informed consent was obtained. This study was conducted in accordance with the principles of the Declaration of Helsinki and reported according to the STROBE guidelines (Table S1).

This cross‐sectional observational study included 141 patients and 50 healthy controls who visited the Division of Oral and Maxillofacial Surgery, Department of Tohoku University Hospital, Miyagi, Japan. The 77 with OPMDs (36 had leukoplakia without dysplasia, and 41 had lichen planus) and 64 with OSCCs (ICD‐11 Codes: 2C61.1–5) were pathologically diagnosed. The sample size was primarily determined by clinical feasibility and participant availability, while ensuring adequate representation across diagnostic categories. Although it is not based on formal power calculations, the sample size is consistent with or exceeds those reported in similar microbiome studies. The pathological diagnosis of OPMD was determined according to the WHO classification of tumors of the oral cavity and mobile tongue (WHO Classification of Tumours Editorial Board 2022). Leukoplakia and oral lichen planus were diagnosed based on clinical and confirmed histopathologically via incisional biopsy according to WHO criteria. Leukoplakia was defined as nonscrapable white plaques, whereas oral lichen planus was defined as bilateral white reticular lesions with characteristic lichenoid inflammation. The 64 patients with OSCCs were referred by their general dental practitioners with chief complaints of white spots, erythema, pain, and discomfort in the oral cavity. None of the patients with OSCC had received chemotherapy or radiotherapy before surgery. Patients with cancer of the lip, tonsils, larynx, or pharynx; those with tumors of origin other than squamous cells; those with recent antibiotic or topical steroid use; and those unable to provide informed consent were excluded. TNM disease stages were obtained from medical records, and patients were classified according to the Union for International Cancer Control system (WHO Classification of Tumours Editorial Board 2022). The OSCC group was divided into an early OSCC group consisting of stages 1 and 2, and an advanced OSCC group consisting of stages 3 and 4. The 50 healthy controls had no prior treatment or oral lesions and no evidence of tumor‐related diseases, oral mucosal diseases, immune diseases, nutritional disorders, or dental infections (Table S2).

Background characteristics attributable to the oral microflora, including age, sex, smoking, history of alcohol consumption, medical history, number of existing teeth, and periodontally affected teeth with a pocket probing depth of 4 mm or more, were investigated at the time of saliva collection. The pocket probing depth was measured for all six remaining teeth, and the greatest depth was adopted as the value; P_Per (%) was defined as the number of teeth with a pocket probing depth of 4 mm or greater/remaining teeth > 100.

### 2.2 Sample Preparation

Unstimulated whole saliva samples (up to 2 mL) were collected in sterile 30 mL polypropylene tubes by directly spitting, prior to any food intake, tooth brushing, oral rinsing, or any form of treatment. All saliva specimens were stored at −60°C immediately after collection and subsequently delivered to Tohoku University Clinical Biobank (Sendai City, Miyagi Prefecture) and frozen at −80°C after aliquoting.

### 2.3 Genomic DNA Extraction

The composition and diversity of the oral microbiome were assessed by high‐throughput sequencing of the 16S rRNA gene amplicons using an Illumina MiSeq platform (Illumina Inc., San Diego, CA, USA). DNA was extracted from the samples using a DNeasy PowerSoil Pro Kit (QIAGEN Inc., Hilden, Germany) according to the manufacturer's protocol. Sequencing libraries were prepared using a two‐step PCR method targeting the V3–V4 hypervariable region of the 16S rRNA gene. PCR amplification was performed using the Takara Ex Taq reagent (TaKaRa Bio Inc., Shiga, Japan). The forward and reverse primers were 5′‐ACACTCTTTCCCTACACGACGCTCTTCCGATCTNNNNNCCTACGGG‐NGGCWGCAG‐3′, and 5′‐GTGACTGGAGTTCAGACGTGTGCTCTTCCGATCTNNNNNGACTACHVGGGTATCTAATCC‐3′, respectively. PCR was performed while setting the temperature at 94°C for 3 min, followed by 30 cycles of 94°C for 30 s, 55°C for 30 s, and 72°C for 30 s. This was followed by an extension period of 72°C for 5 min and a final hold at 4°C. Thereafter, 3 μL of the 20 μL amplification product and 2% agarose gel were employed to ascertain the presence of PCR bands, which were identified at around 540 bps.

Subsequently, a second PCR was conducted using distinct indexing primers that integrated Illumina sequencing adapters and dual barcodes into the amplicon. The pooled library was quantified using a Qubit 2.0 Fluorometer and dsDNA HS Assay Kit (Life Technologies, Carlsbad, CA, USA), before being diluted to a final concentration of 12 pM with 50% PhiX. Sequencing was conducted using a MiSeq Reagent Kit v3 (Illumina Inc.) with a paired‐end sequencing protocol in accordance with the manufacturer's instructions. In total, 2.7 million paired‐end reads were generated. The mean read pair count for the samples was 24,065, with a maximum of 82,113 read pairs.

### 2.4 Amplicon Sequence Variants (ASVs)

Sequence data for the 16S rRNA gene amplicons were analyzed using the QIIME2 platform, version 2024.2 (Bolyen et al. 2019; Caporaso et al. 2010). The initial 20 bases of both sequences were excised to remove the primer sequences for all paired reads. Bases subsequent to position 280 were truncated to remove low‐quality sequence data, and potential amplicon sequencing errors were corrected using DADA2 to generate an ASV dataset (Katoh and Standley 2013). The ASV results were aligned using the MAFFT software (version 7.526) (Katoh and Standley 2013) and were subsequently used to construct a phylogenetic tree using FastTree2 (Price et al. 2010). Alpha‐ and beta‐diversity metrics were estimated from a subsampled ASV dataset, using 1000 sequences per sample. Each ASV was identified using a Naïve Bayes classifier trained on the 16S rRNA gene sequences from the Greengenes2 database (McDonald et al. 2024). Fifteen ASVs were assigned to the phylum, 19 to the class, 191 to the genus, and 386 to the species levels.

The observed richness was defined as the number of different taxa in a community and was the simplest alpha‐diversity metric. The Shannon diversity index is a widely used alpha diversity metric and can be considered the uncertainty of whether two random individuals in a sample/community are similar (Shannon 1948). If all taxa had the same abundance, then the evenness was high; if one or a few taxa dominated, the evenness was low. Faith's phylogenetic distance (Faith PD) incorporates the phylogenetic tree of the taxa and is the sum of all branch lengths that connect all taxa observed in the sample (Faith 1992). The β‐diversity was evaluated using UniFrac principal coordinates‐based principal coordinate analysis (PCoA) (Gower 1966). The data were projected onto two‐dimensional space using principal components 1 (PCoA1) and 2 (PCoA2). Clusters of different colors represent separate groups, allowing for the visualization of similarities between samples and differences between groups. The mean PCoA values for each group along the x‐ and y‐axes are indicated by circular shapes, and the mean and standard error are shown as representative values.

The microbiome data is represented by the relative abundance at each taxonomic level, and comparisons between groups were performed for all observed bacteria.

### 2.5 Statistical Analysis

Statistical analyses were conducted using GraphPad Prism and JMP 17.0. The Steel–Dwass test was employed as a nonparametric test to perform three or more comparisons among groups. The differential abundance of genera among groups was identified using analysis of microbiome compositions with the bias correction (ANCOM‐BC) method. Statistical significance was set at *p* < 0.05.

## 3 Results

### 3.1 Patient Characteristics

The clinical characteristics of healthy controls and patients with leukoplakia, lichen planus, and OSCC are shown in Table S3. According to the TNM staging system, 15, 28, 8, and 13 patients were classified as having stage I, II, III, and IV oral cancers, respectively. No patient in the control group was diagnosed with OSCC or OPMDs at any site. During the follow‐up period (12–48 months), no patient in the control, leukoplakia, and lichen planus groups developed OSCC.

### 3.2 Alpha Diversity

**FIGURE 1:**
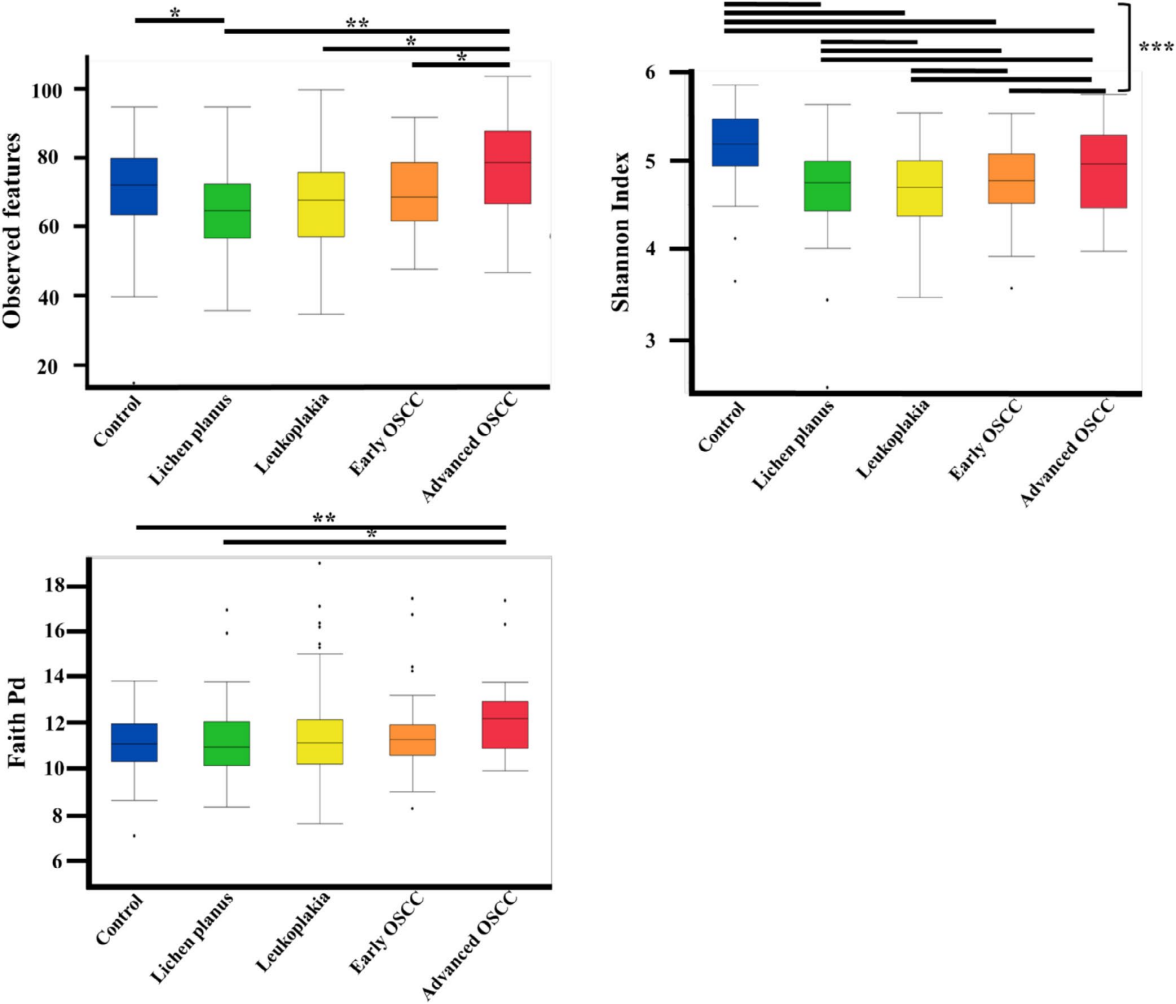
Richness is the number of pure species found in the saliva of each group. The Shannon diversity index, an alpha diversity metric, is widely used as a measure of information and can be thought of as the uncertainty regarding whether two random individuals in a sample/community are similar. If all taxa have the same abundance, evenness is high, and if one or few taxa dominate, evenness is low. Faith's PD is an alpha diversity metric that incorporates the phylogenetic tree of the taxa. Faith's PD is the sum of all the branch lengths connecting all taxa observed in the sample. Box plots of the observed features, Shannon entropy, and Faith's PD in the saliva of each group are shown. The observed features indicated the index of all detected operational taxonomic units. Shannon entropy indicates the evenness of species in each community. Faith's PD indicates the diversity index calculated based on the phylogenetic tree. *p < 0.05, **p < 0.01, and ***p < 0.01 show significant differences calculated using the Steel–Dwass test.

In total, 275 species were identified at the genus level in the saliva samples, and alpha‐diversity indices were calculated (Figure 1, Table S4). Alpha‐diversity metrics revealed differences in the richness, evenness, and phylogenetic breadth of the salivary microbiome across groups. The average number of observed bacterial genera (richness) was highest in the advanced OSCC group (median = 79.0), followed by the control (72.5), early OSCC (68.0), leukoplakia (65.0), and lichen planus (69.0) groups (Table S4). The standard deviation for richness was notably higher in the advanced OSCC group (14.62), indicating greater inter‐individual variability in microbial diversity.

The Shannon entropy, which measures both richness and evenness, was significantly higher in the control group (median = 5.18) than in the OSCC groups, suggesting that microbial communities in cancer patients are more unevenly distributed. Faith's phylogenetic diversity (PD), reflecting the evolutionary breadth of the microbial community, was significantly greater in advanced OSCC (median = 12.25), indicating a broader range of microbial lineages.

Among OSCC patients, those with poor histopathological differentiation or clinical lymph node metastasis (N1 or higher) had significantly higher Faith's PD values. No significant differences in Faith's PD were observed according to T classification, stromal lymphocytic reaction, mode of invasion, or depth of invasion, although values tended to be higher in cases with higher clinicopathologic malignancy grade.

### 3.3 Beta Diversity

**FIGURE 2:**
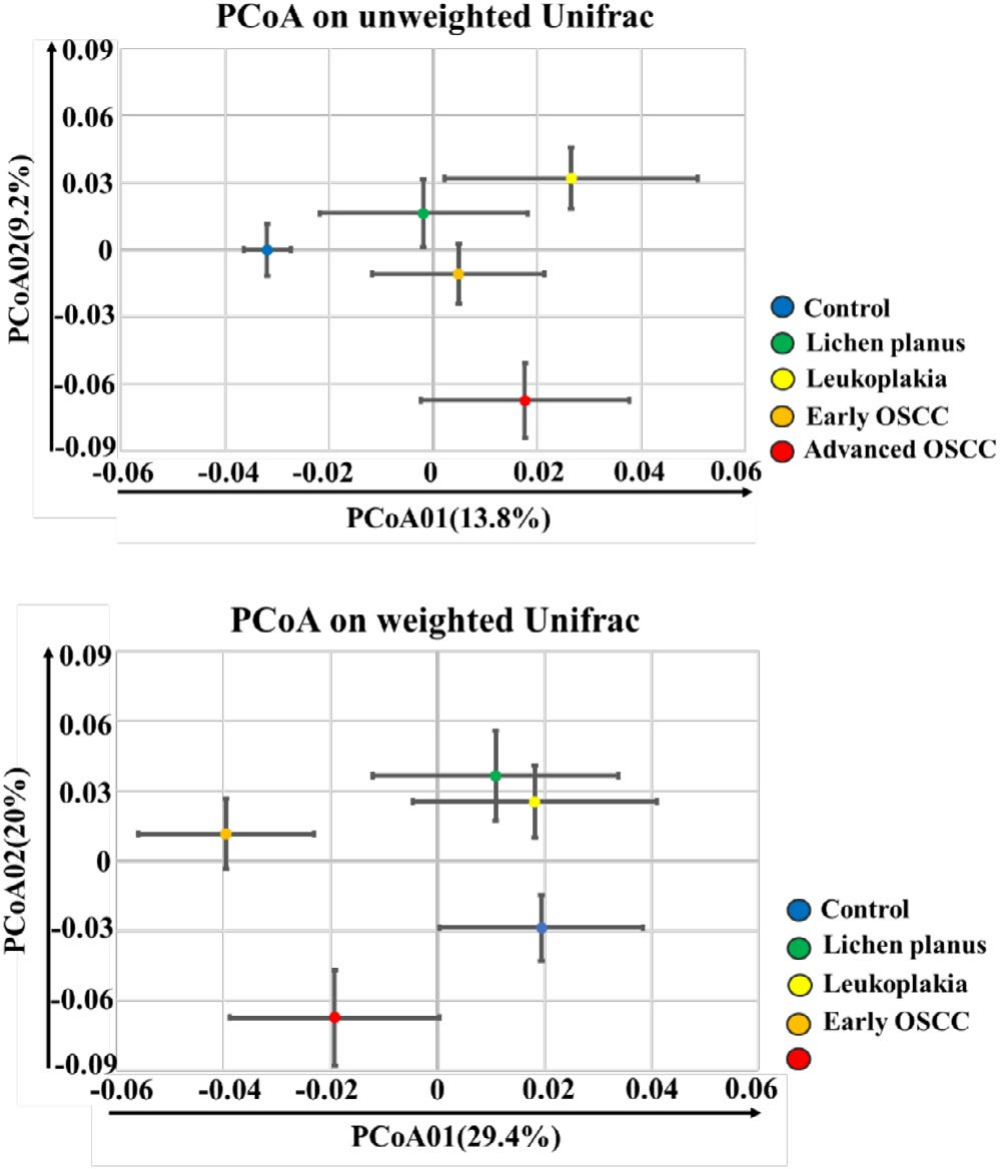
PCoA. Each point represents a sample, with the data being projected onto a two‐dimensional space by PCoA1 and PCoA2. Clusters in different colors represent different groups which help visualize similarities between samples and the separation between groups. Weighted Unifrac: Lead count is considered (evaluated with lead count weight), Unweighted Unifrac: Lead count is not considered. Each plot in 2‐dimensional PCoA based on weighted and unweighted UniFrac distances shows the mean values of the principal coordinates in each category classified by pathological diagnosis. The bars indicate standard divisions. Weighted PCoA considered the read count. Unweighted PCoA did not consider the read count.

The beta diversity was evaluated using UniFrac principal coordinate‐based PCoA (Figure 2, Table S5). PCoA using UniFrac distances showed distinct microbial clustering patterns associated with disease progression. In the unweighted PCoA, advanced OSCC samples were clearly separated from the other groups along the PCoA2 axis (average = −0.06732), whereas early OSCC and controls were more closely clustered near the origin (Table S5). In the weighted PCoA, both early and advanced OSCC groups were distinctly separated along the PCoA1 axis (average = −0.03953 and −0.01927, respectively), indicating alterations in microbial composition and abundance with disease progression.

Additionally, weighted and unweighted PCoAs demonstrated that OSCC patients with deeper submucosal invasion, larger T3/T4 tumors, and lymph node metastasis (N1 or greater) formed independent clusters, indicating substantial shifts in microbiota structure associated with advanced clinical and pathological features.

### 3.4 Class‐Level Microbiome

**FIGURE 3:**
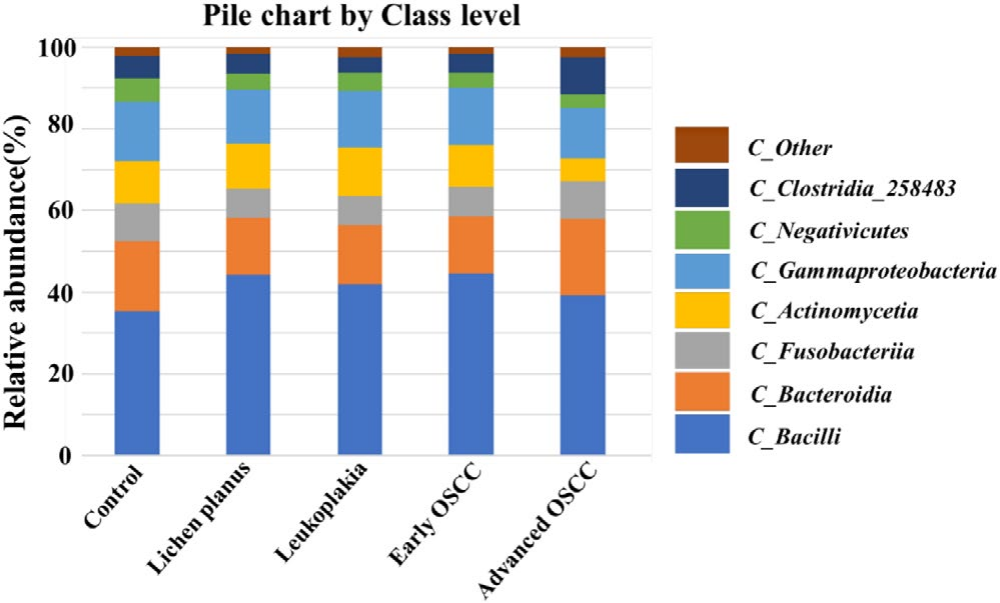
Stacked bar graphs focusing on the microbiome at the class level. The relative abundances of different bacterial taxa in each group are shown. Each color corresponds to a different bacterial group, and the height of each colored area represents the percentage of the bacterial group in the sample from each site. Stacked bar plots and relative abundances of major bacterial species (> 1%) at the class level in each diagnosed category are shown. Less than 1% of the species were classified as ‘other’.

**FIGURE 4:**
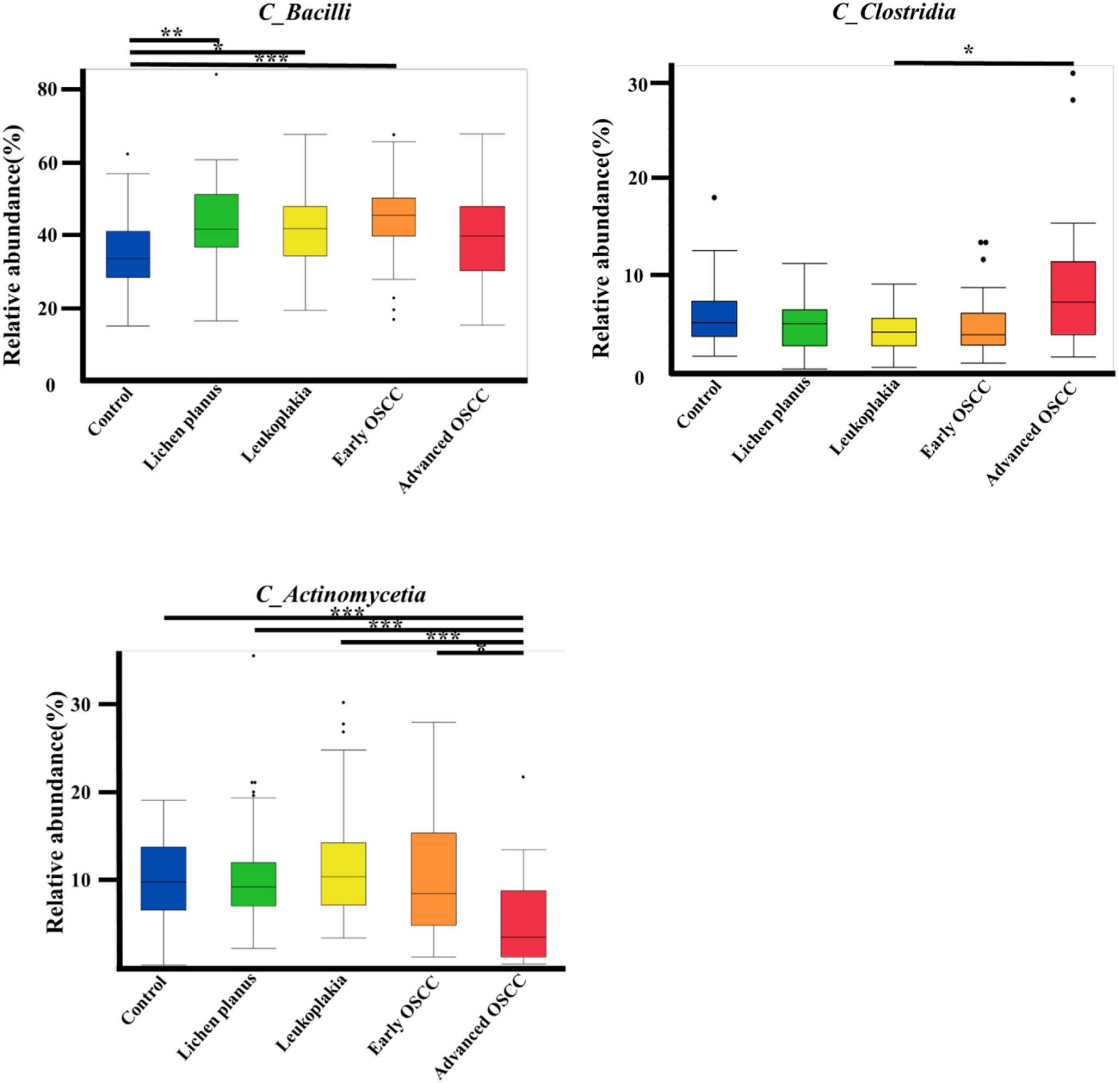
Relative abundance of *Actinomycetia* and *Bacilli* at the class level among the five groups. Box plots of relative abundance of *Actinomycetia*, *Bacilli*, and *Clostridia* among the five groups are shown. The Steel–Dwass test revealed a significant difference between the groups for the two bacterial classes. *p < 0.05, **p < 0.01, and ***p < 0.001 show significant differences calculated using the Steel–Dwass test.

Stacked bar graphs focusing on the microbiome class for each group are shown in Figure 3. Steel–Dwass tests were performed to identify significantly higher or less than three bacterial taxa in the five groups (Figure 4, Table S6).

Quantitative analysis of class‐level relative abundances supported the presence of microbial shifts associated with disease status. The abundance of *Bacilli* was highest in the early OSCC group (mean = 44.60%) and lowest in the controls (mean = 35.27%), showing a progressive increase from health to premalignancy to malignancy (Table S6). *Actinomycetia* were more prevalent in the leukoplakia (mean = 11.91%) and lichen planus groups (mean = 11.01%) than in the advanced OSCC group (mean = 5.53%), indicating a potential association with premalignant conditions. *Clostridia* were significantly elevated in the advanced OSCC group (mean = 9.04%) compared to all other groups.

Furthermore, class‐level analysis stratified by clinical and histopathological grades revealed that *Bacilli* were consistently elevated in the OSCC group regardless of grading category, while *Actinomycetia* and *Negativicutes* were significantly reduced. *Clostridia* were notably enriched in OSCC cases with lymph node metastasis (N1 or higher), whereas *Actinomycetia* were significantly decreased in OSCC patients with large tumors (T3 and T4) compared to those with smaller tumors (T1 and T2).

### 3.5 Heat Map

**FIGURE 5:**
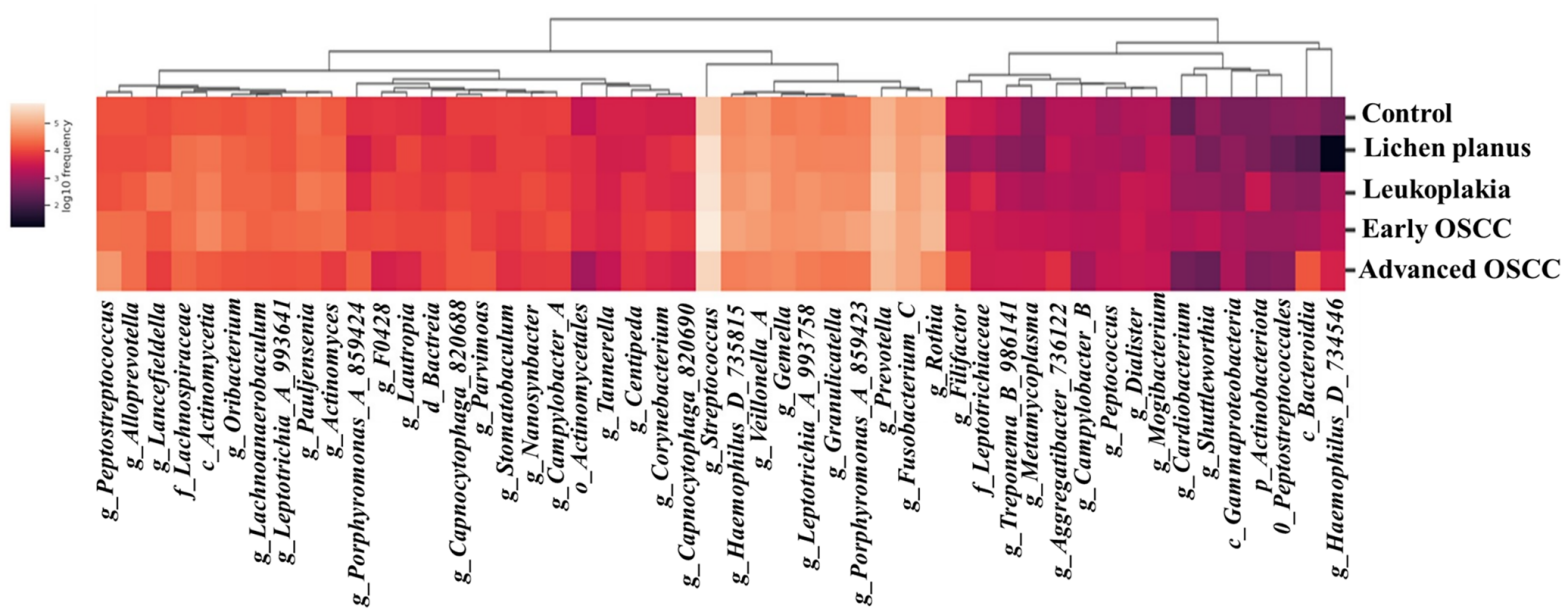
Heat map of the salivary microbiome composition of each group. A heat map was created to depict the abundance of each bacterial species at the genus level. Darker orange indicates more abundant species, and darker purple indicates less abundant species. This optimized heat map illustrates the microbial features selected and ranked using machine learning to predict the characteristics of the salivary microbiomes in the five groups. Each column represents a microbial feature at the genus level, and the rows correspond to each group. The color gradient corresponds to the log_10_ frequency of the abundance, with darker colors representing lower bacterial clustering shown at the top of the dendrogram.

A heat map was generated to visualize the relative abundance of major bacterial genera across the five study groups (Figure 5). Each column represents a genus‐level taxon, and each row corresponds to an individual subject grouped by clinical diagnosis. The color intensity indicates the log10‐transformed abundance, with orange hues representing higher abundance and purple hues indicating lower abundance.

Hierarchical clustering was applied to both genera and samples to identify microbial similarity. Notably, samples from the advanced OSCC group formed a distinct cluster, characterized by elevated levels of *Peptostreptococcus*, *Fusobacterium*, and *Dialister*, and reduced levels of commensal genera such as *Streptococcus* and *Rothia*. Conversely, samples from the control and OPMD groups were more heterogeneous but tended to cluster together, reflecting similar microbial compositions with predominance of *Actinomycetia*, *Veillonella*, and *Streptococcus*.

These results suggest a gradual microbial shift from healthy mucosa to OPMDs and finally to OSCC, with specific genera such as *Fusobacterium* and *Peptostreptococcus* becoming more prominent in the cancerous state. The distinct clustering observed in advanced OSCC highlights the potential utility of salivary microbial profiling in stratifying disease stages.

### 3.6 ANCOM‐BC

**FIGURE 6:**
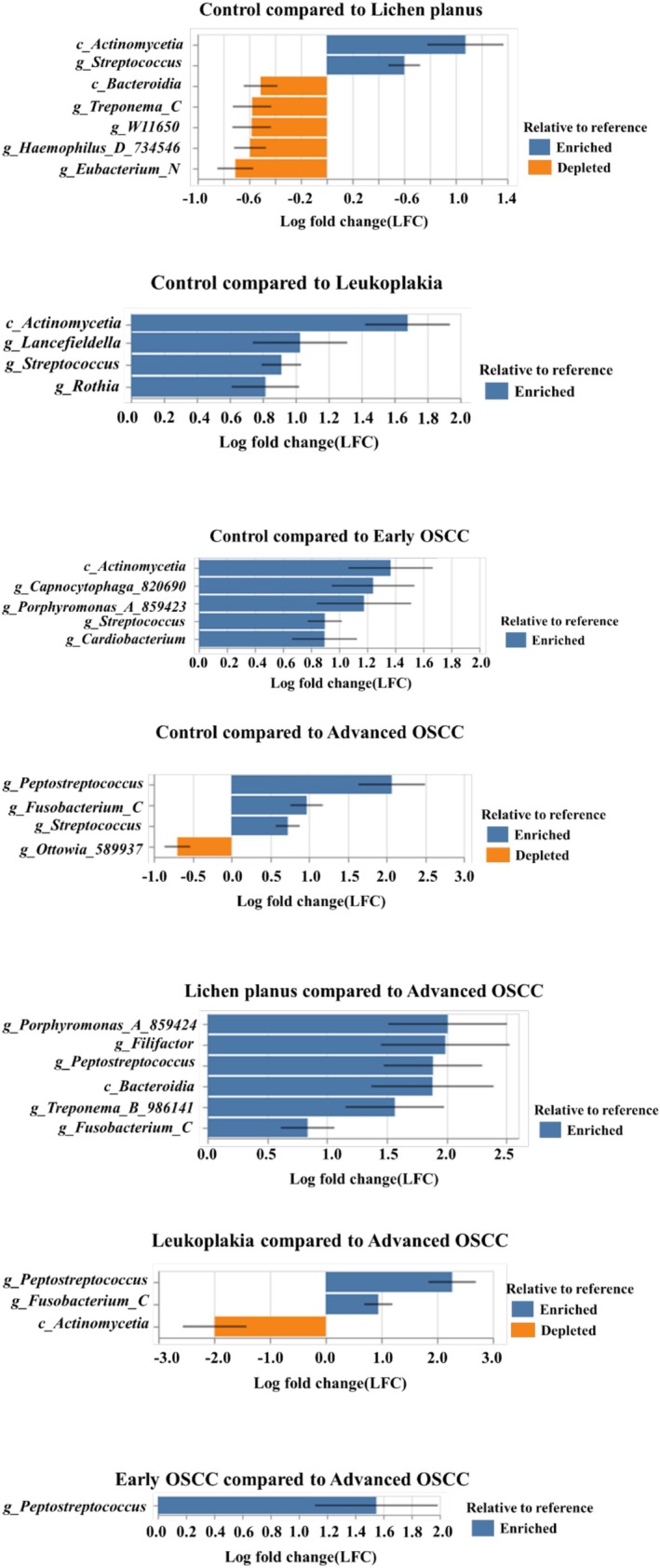
ANCOM‐BC method for differential abundance testing of samples between groups. ANCOM‐BC of the salivary microbiome was performed between each clinical characteristic and pathological grade. Several bacterial species at the genus level showed significant increases or decreases in abundance between groups. The effect size (log‐fold change) with the standard error and 95% confidence interval obtained using the ANCOM‐BC package are shown. Positively and negatively altered pathways are indicated by pale blue and pale yellow bars, respectively.

In total, 275 species were identified at the genus level. The differentially abundant taxa were examined among the five groups (Figure 6). ANCOM‐BC was used to test for differences between samples from each group and identify bacteria that were significantly more or less abundant (*p* < 0.05). Compared with the control group, *Actinomycetia*, *Streptococcus*, *Lancefieldella*, and *Rothia* were significantly elevated in the leukoplakia group; *Actinomycetia* and *Streptococcus* were significantly elevated in the lichen planus group; and *Actinomycetia*, *Streptococcus*, *Capnocytophaga*, *Porphyromonas*, and *Cardiobacterium* were significantly elevated in the early OSCC group. Seven species showed a significant increase in *Peptostreptococcus*, *Bacteroidia*, *Fusobacterium*, and *Streptococcus* in the advanced OSCC group compared to the other groups. Of these, *Peptostreptococcus* and *Fusobacterium* were significantly elevated in the advanced OSCC group compared to the control, leukoplakia, lichen planus, and early OSCC groups.

## 4 Discussion

In this study, the authors aimed to clarify the bacteria associated with the development and suppression of oral cancer and OPMDs, and discovered characteristic changes in the bacterial flora during progression from OPMDs to early OSCC, followed by advanced OSCC.

Leukoplakia is characterized by white patches not attributable to known causes, and an unclear underlying mechanism (van der Waal et al. 1997; Warnakulasuriya et al. 2007, 2021). The relationship between leukoplakia and infection caused by pathogenic microorganisms has long been focused on HPV, which has a strong affinity for the epithelium, with more than 100 HPV types being reported. Some HPV types are associated with cervical cancer, precancerous lesions of the cervix, and pharyngeal carcinoma (Campisi et al. 2004; Toki et al. 1986). Mizuki (2001) reported the infection of leukoplakia epithelial cells with *Mycoplasma* spp. Furthermore, Amer et al. (2017) observed an increase in *Fusobacterium* and *Candida* spp. and a decrease in *Firmicutes* spp. in the oral cavities of patients with oral leukoplakia. Moreover, the bacterial flora detected in patients with oral leukoplakia are similar to those detected in colorectal cancer, especially coexisting *Fusobacterium*, *Leptothrix*, and *Campylobacter* spp. (Hu et al. 2016).

Oral lichen planus is a chronic mucocutaneous disease, typically presenting with bilateral, symmetrical white striations, papules, or plaques, often accompanied by erythema or erosions. This disease is diagnosed based on clinical and histopathological criteria as an OPMD due to its malignant potential (Warnakulasuriya et al. 2021). Oral lichen planus has a T‐cell‐mediated autoimmune pathogenesis, wherein antigens, possibly presented by basal keratinocytes, trigger cytotoxic T‐cell responses, activating CD8^+^ T cells to induce epithelial apoptosis. Additionally, CD4^+^ T cells and cytokines such as IL‐17 (Solimani et al. 2019) and TNF‐α (Mozaffari et al. 2019) amplify the inflammatory response, maintaining chronicity. According to Wang et al. (2015), the interactions between microorganisms and immunity may play direct or indirect roles in the development of oral lichen planus. They found that *Porphyromonas* and *Prevotella melaninogenica* were predominant in erosive and reticulate oral lichen planus, respectively. Linkers of *Streptococcus* species are significantly less common in erosive oral lichen planus, suggesting the importance of *Streptococcus* in the pathogenesis of oral lichen planus. Some genera, such as *Porphyromonas*, were associated with disease severity and immune dysregulation.

Alpha‐diversity analysis showed that the total number of bacterial species and Shannon Index were high in the advanced OSCC and control groups. This implies that, with OPMD onset in healthy oral mucosa, the richness and heterogeneity of the bacterial flora in the saliva sample decreased and then increased as the cancer progressed and matured. Faith's PD, which is the total number of branches of the phylogenetic tree of bacterial species, was significantly higher only in advanced OSCC. Therefore, more complex and widespread bacterial species were present in the oral cavity after carcinogenesis. Additionally, beta‐diversity analysis showed that clusters of healthy mucosae in the control group, clusters of OPMDs such as leukoplakia and lichen planus, and the cluster of early OSCC were all close, despite some differences. However, only advanced OSCC showed a distinctive degree of diversity. Particularly, the results showed that the number of *Bacillus* species in the OPMD group was increased when comparing mucosal changes between healthy patients and patients with OPMDs. The pathogenicity of *Bacillus* spp. is mediated by various factors including extracellular enzymes (phospholipase, protease, and chitinase), cytotoxic proteins (haemolysin, enterotoxin, and cytotoxin), and cell surface proteins (Ehling‐Schulz et al. 2019). These factors are involved in host adhesion, nutrient acquisition, and evasion of the host immune response. Although the involvement of these bacterial biochemical characteristics in oral cancer, other solid cancers, or OPMDs has not been reported, *B. cereus* is increasingly being recognized as a causative agent of localized wound infections, eye infections, and systemic infections that affect oral mucosal changes in OPMD (Bottone 2010). Additionally, *Bacillus* spp. may be involved in the activation of immune cells and enhancement of antitumour immune responses by altering the tumor microenvironment (Pushalkar et al. 2018).

The *Actinomycetia* genus, a common oral bacterium, is a particularly notable species that was significantly increased in the leukoplakia, lichen planus, and control groups compared to that in the early stage and advanced OSCC groups. *Actinomycetia* produce membrane vesicles that induce mitochondrial dysfunction and reactive oxygen species production in colorectal epithelial cells, causing DNA damage and possibly promoting the development of colorectal cancer (Breau 2024). However, to date, no study has reported similar changes in the OPMDs. Nonetheless, the enrichment of these bacteria correlates with precancerous conditions and early stages of gastric carcinogenesis; thus, they may serve as noninvasive and accurate screening tools for early‐stage gastric cancer (Zhou et al. 2022). Likewise, in the present study, the *Streptococcus* genus was clearly increased in the OPMD groups, compared to that in the control group. Therefore, *Streptococcus*, which is abundant in the oral cavity, may be used as a screening tool for premalignant or early cancerous lesions.

Furthermore, the results of this study showed that the number of *Clostridia*, *Porphyromonas*, and *Fusobacterium* species was significantly increased in the advanced OSCC group, suggesting their involvement in cancer progression. Clinical evidence has also shown that *C. symbiosum* , a species of *Clostridia*, is enriched in tumor tissues from patients with colorectal cancer and stool samples from patients with adenoma recurrence after endoscopic colorectal polypectomy (Roelands et al. 2023), whereas studies using mouse models have shown that *C. symbiosum* promotes colorectal tumorigenesis and tumor growth. Additionally, this bacterium has been shown to enhance cell proliferation and stem cell properties by producing branched‐chain amino acids and activating cholesterol synthesis and Hedgehog signaling, which are mechanisms involved in carcinogenesis (Ren et al. 2024). The authors of the present study previously used an in vitro invasion assay to determine whether *Porphyromonas somerae* can invade endometrial adenocarcinoma cells (Crooks et al. 2021). Their results showed that *Porphyromonas* shares mechanisms for intracellular invasion and persistence and that both species exhibit fumarate‐ and succinate‐driven energy production, intracellular capsule polysaccharide production, and endopeptidase gene components, suggesting their involvement in endometrial cancer progression. Among the *Fusobacterium* species, *F. nucleatum* has been implicated in colorectal cancer in recent years, along with bacteria such as *Escherichia coli* , enterotoxin‐producing *Bacteroides fragilis* , and pks + *E. coli* (Rubinstein et al. 2013). *Fusobacterium nucleatum* is thought to cause colorectal cancer by adhering to colorectal epithelial cells, Toll‐like receptor activation, nuclear factor kappa B signaling, reactive oxygen species induction, and ultimately, the development of mutagenic DNA damage, which promotes tumor progression via pathogenic mechanisms.

Based on the results of previous studies and the current study, if these bacterial communities prove to be involved in the malignant transformation of the oral mucosa, it may be possible to identify patients at high risk of developing OSCC and prevent malignant transformation through local antibiotic therapy. Consequently, the bacterial flora in saliva could function as a biomarker for oral cancer, allowing us to accurately identify the signs of cancer development and provide effective treatment at an early stage, if the authors can capture the changes in the bacterial flora that occur at the precancerous and early cancer stages.

A strength of this study is its larger sample size compared with previous studies involving bacterial flora analysis of oral cancer saliva. Recent studies have shown that the salivary microbiome differs between healthy individuals, patients with epithelial dysplasia, and those with OSCC. Radaic et al. (2024) reviewed key bacterial genera that may contribute to inflammation and immune modulation in OPMDs and OSCC, such as *Fusobacterium*, *Porphyromonas*, and *Prevotella*. Galvin et al. (2025) reported reduced alpha diversity and distinct bacterial profiles across disease stages, highlighting the potential diagnostic value of salivary microbiota. Our results are consistent with these findings, supporting the idea that oral microbial dysbiosis is associated with progression from OPMDs to OSCC. Unlike previous studies, our analysis included a Japanese cohort and focused specifically on 16S rRNA‐based profiling, adding to the current understanding of regional and population‐level variations. Nonetheless, the study has some limitations. First, due to its cross‐sectional design, causal relationships between microbiota alterations and disease progression cannot be established; our results indicate association rather than causation. Second, the sampling was conducted at a single institution in northeastern Japan, which may limit the generalizability of the findings to other populations. Finally, we did not compare the oral bacterial flora of OSCC with that of other OPMDs, such as oral erythroplakia or actinic cheilitis; therefore, future studies investigating this aspect are warranted. Although this study was a cross‐sectional study, the authors believe that longitudinal studies that observe the long‐term course of patients with OPMD and classify them into groups that develop cancer and groups that do not are necessary. Furthermore, the authors believe that it is necessary to conduct biochemical analyses of the bacterial species identified as being involved in carcinogenesis or cancer progression and to verify this using animal experiments in which specific bacteria are investigated.

In conclusion, the study findings suggest that the composition and diversity of oral microbiota are associated with OPMD development and progression to OSCC. Therefore, the salivary microbiome may serve as a biomarker for oral cancer and help predict cancer progression.

## Author Contributions

**Atsumu Koketsu:** conceptualization, methodology, funding acquisition, writing – original draft, project administration. **Satoshi Fukase:** software, validation, investigation, visualization. **Toru Tamahara:** conceptualization, formal analysis, writing – review and editing, investigation, validation. **Tatsuru Saito:** validation, investigation. **Akiko Ito:** investigation, validation. **Yutaro Higashi:** validation, investigation. **Tomonari Kajita:** investigation, validation. **Tsuyoshi Kurobane:** investigation, validation. **Masaaki Miyakoshi:** investigation, validation. **Masahiro Iikubo:** supervision, writing – review and editing, conceptualization. **Kazuki Kumada:** supervision, conceptualization, writing – review and editing. **Bin Li:** conceptualization, supervision, writing – review and editing. **Muneaki Shimada:** conceptualization, writing – review and editing, supervision. **Ritsuko Shimizu:** conceptualization, project administration, writing – review and editing. **Tetsu Takahashi:** writing – review and editing, supervision, project administration. **Kensuke Yamauchi:** writing – review and editing. **Tsuyoshi Sugiura:** writing – review and editing, project administration.

## Ethics Statement

The study protocol was approved by the Ethics Committee of Tohoku University Graduate School of Dentistry (No. 25091).

## Consent

The study design and objectives were explained to the patients, and written informed consent was obtained.

## Conflicts of Interest

The authors declare no conflicts of interest.

## Supporting information


**Table S1:** STROBE Checklist.

**Table S2:** Inclusion and exclusion criteria.

**Table S3:** Clinicopathological features of each group.

**Table S4:** Alpha‐diversity metrics. Richness is the number of pure species found in the saliva samples from each group.

**Table S5:** Beta diversity evaluated using PcoA.

**Table S6:** Relative abundance of *Bacilli*, *Actinomycetia* and *Clostridia* at the class level among five groups.

## Data Availability

The datasets generated and/or analyzed during the current study are not publicly available due to the included personal patient information but are available from the corresponding author upon reasonable request.
