# Supplementary material for: Bacterial Involvement in Oral Squamous Cell Carcinoma and Potentially Malignant Oral Disorders
Source: Oral Dis. 2025 Oct 9;32(4):992–1003. doi: 10.1111/odi.70115 (PMC13248574; doi:10.1111/odi.70115)
Supplement: Supplementary file 1 — Table S1: STROBE Checklist. [file ODI-32-992-s001.docx]

# S1: STROBE Checklist

| Item No | Recommendation | Page No | Relevant Text |
| --- | --- | --- | --- |
| 1 (a) | Indicate the study’s design with a commonly used term in the title or abstract | 1 | Title: “Bacterial involvement in oral squamous cell carcinoma and potentially malignant oral disorders”; Abstract: “This cross-sectional observational study …” |
| 1 (b) | Provide an informative and balanced summary in abstract | 1 | Summarizes background (OSCC/OPMD & microbiota), methods (16S rRNA metagenomic), results, conclusion. |
| 2 | Explain the scientific background and rationale | 2 | Introduction outlines OSCC risk factors and gap in OPMD progression microbiome studies. |
| 3 | State specific objectives and hypotheses | 2 | “Aim to clarify relationship between oral microbiota and OPMD/OSCC progression via metagenomic analysis.” |
| 4 | Present key elements of study design early | 4 | Described as cross-sectional observational study with controls, OPMD, OSCC groups. |
| 5 | Describe setting, locations, dates | 4 | Saliva collected at Tohoku University Hospital before treatment, stored at –80℃. |
| 6 (a) | Give eligibility criteria and selection methods | 4–5 | Inclusion: histopathologic OPMD/OSCC, controls; Exclusion: other tumors, antibiotic/steroid use, etc. |
| 6 (b) | Matching criteria (n/a) | - | Not a matched study design. |
| 7 | Define outcomes, exposures, confounders | 5 | Outcomes: microbiota composition/diversity; Exposure: disease status; Confounders: age, sex, smoking, alcohol, dentition. |
| 8 | Data sources and assessment methods | 5–6 | Unstimulated saliva; 16S rRNA V3–V4 sequencing; QIIME2 pipeline. |
| 9 | Describe efforts to address bias | 6 | Excluded antibiotic/steroid users; documented confounders. |
| 10 | Explain how study size was arrived at | 6 | 191 participants based on feasibility; aligns with similar studies. |
| 11 | Explain handling of quantitative variables | 7 | Richness, Shannon, Faith PD as continuous variables; relative abundances. |
| 12 (a) | Describe all statistical methods | 7–8 | Steel–Dwass, ANCOM-BC, QIIME2 diversity analyses, p<0.05. |
| 12 (b) | Methods for subgroups and interactions | 8 | Subgroup analyses by TNM stage and histopathology. |
| 12 (c) | Explain how missing data were addressed | 8 | Minimal missing data; detailed in Table S3. |
| 12 (d) | Loss to follow-up/matching (n/a) | - | Cross-sectional design; not applicable. |
| 12 (e) | Describe sensitivity analyses | 8 | Additional PCoA and heatmap clustering. |
| 13 (a) | Report numbers at each stage | 10 | 50 controls, 77 OPMD, 41 early OSCC, 20 advanced OSCC. |
| 13 (b) | Reasons for non-participation | 5 | Exclusion criteria. |
| 13 (c) | Flow diagram | - | Not included. |
| 14 (a) | Participant characteristics | 10–11 | Age, sex, smoking, alcohol, dental status (Table S3). |
| 14 (b) | Missing data counts | 10, S3 | Footnotes in Table S3. |
| 14 (c) | Follow-up time summary | 10 | Follow-up 12–48 months; no malignant progression in controls/OPMD. |
| 15 | Outcome data reporting | 12–14 | Diversity indices, PCoA clusters, taxonomic abundances. |
| 16 (a) | Unadjusted and adjusted estimates | 12–14 | Steel–Dwass p-values; ANCOM-BC log-fold changes with 95% CI. |
| 16 (b) | Category boundaries | - | Not applicable (continuous variables). |
| 16 (c) | Translate relative to absolute risk | - | Not applicable. |
| 17 | Other analyses | 14–16 | Heatmap (Fig.5), PCoA (Fig.2), ANCOM-BC (Fig.6). |
| 18 | Summarise key results | 19 | Discussion highlights microbiota shifts and biomarker potential. |
| 19 | Discuss limitations | 20 | Cross-sectional, single-center, limited OPMD types. |
| 20 | Cautious interpretation | 21 | Linked findings to prior studies; called for longitudinal research. |
| 21 | Discuss generalisability | 21 | Population-specific considerations; diet/hygiene context. |
| 22 | Source of funding and role | 2 | Supported by JSPS KAKENHI 22K17150; no funder involvement. |
