# Supplementary material for: Bacterial Involvement in Oral Squamous Cell Carcinoma and Potentially Malignant Oral Disorders
Source: Oral Dis. 2025 Oct 9;32(4):992–1003. doi: 10.1111/odi.70115 (PMC13248574; doi:10.1111/odi.70115)
Supplement: Supplementary file 2 — Table S2: Inclusion and exclusion criteria. [file ODI-32-992-s003.docx]

Table S2. Inclusion and exclusion criteria

| **Criteria** | **Details** |
| --- | --- |
| Inclusion Criteria | Patients with histopathologically diagnosed OPMDs (leukoplakia or lichen planus) |
| Inclusion Criteria | Patients with histopathologically diagnosed OSCC (stage 1–4) |
| Inclusion Criteria | Patients or healthy volunteers capable of providing saliva samples |
| Inclusion Criteria | Healthy individuals with no clinical signs of oral mucosal disease |
| Inclusion Criteria | Individuals who provided written informed consent |
| Exclusion Criteria | Patients with cancers originating from the lips, tonsils, pharynx, or larynx |
| Exclusion Criteria | Patients with tumors of non-squamous cell origin |
| Exclusion Criteria | Individuals who had recently used antibiotics or topical steroids |
| Exclusion Criteria | Patients with a history of chemotherapy or radiotherapy for OSCC |
| Exclusion Criteria | Individuals unable to provide informed consent |
