# Supplementary material for: Bacterial Involvement in Oral Squamous Cell Carcinoma and Potentially Malignant Oral Disorders
Source: Oral Dis. 2025 Oct 9;32(4):992–1003. doi: 10.1111/odi.70115 (PMC13248574; doi:10.1111/odi.70115)
Supplement: Supplementary file 3 — Table S3: Clinicopathological features of each group. [file ODI-32-992-s005.docx]

Table S3. Clinicopathological features of each group

| **Variables** |  | Control (N=50) | | Lichen planus (N=36) | | Leukoplakia (N=41) | | Early OSCC (N=43) | | Advanced OSCC (N=21) | | p-value |
| --- | --- | --- | --- | --- | --- | --- | --- | --- | --- | --- | --- | --- |
|  |  |  |  |  |  |  |  |  |  |  |  |  |
| **Age** | Years | 60.3 ± 12.4 | | 63.8 ± 11.7 | | 63 ± 11.8 | | 62 ± 16.6 | | 67.9 ± 12.6 | | N.S. |
|  |  |  |  |  |  |  |  |  |  |  |  |  |
| **Sex** | Male | 20 | | 9 | | 24 | | 20 | | 11 | | 0.044 |
|  | Female | 30 | | 27 | | 17 | | 23 | | 10 | |  |
|  |  |  |  |  |  |  |  |  |  |  |  |  |
| **Drinker** | Current | 19 | | 12 | | 15 | | 16 | | 12 | | 0.605 |
|  | Never | 31 | | 17 | | 18 | | 26 | | 9 | |  |
|  | Unknown | 0 | | 7 | | 8 | | 1 | | 0 | |  |
|  |  |  |  |  |  |  |  |  |  |  |  |  |
| **Smoker** | Current | 9 | | 6 | | 14 | | 6 | | 8 | | 0.057 |
|  | Previous | 6 | | 3 | | 5 | | 9 | | 4 | |  |
|  | Never | 35 | | 21 | | 14 | | 27 | | 9 | |  |
|  | Unknown | 0 | | 6 | | 8 | | 1 | | 0 | |  |
|  |  |  |  |  |  |  |  |  |  |  |  |  |
| **Diabetes** | Current | 7 | | 3 | | 4 | | 8 | | 3 | | 0.676 |
|  | Never | 43 | | 33 | | 37 | | 35 | | 18 | |  |
|  |  |  |  |  |  |  |  |  |  |  |  |  |
| **Hyperlipidemia** | Current | 12 | | 9 | | 7 | | 8 | | 4 | | 0.878 |
|  | Never | 38 | | 27 | | 34 | | 35 | | 17 | |  |
|  |  |  |  |  |  |  |  |  |  |  |  |  |
| **Hypertension** | Current | 22 | | 18 | | 19 | | 19 | | 15 | | 0.263 |
|  | Never | 28 | | 18 | | 22 | | 24 | | 6 | |  |
|  |  |  |  |  |  |  |  |  |  |  |  |  |
| **Tooth** |  | 23.8 ± 5.5 (N=50) | | 22.9 ± 7.3 (N=36) | | 23.7 ± 5.9 (N=40) | | 21.7 ± 7.9 (N=41) | | 20.4 ± 8.5 (N=20) | | N.S. |
|  |  |  |  |  |  |  |  |  |  |  |  |  |
| **P_Per (%)** |  | 21.7 ± 16.8 (N=50) | | 19.6 ± 18.8 (N=36) | | 22.5 ± 14.3 (N=39) | | 29.1 ± 26.1 (N=40) | | 39.7 ± 28.6 (N=20) | | N.S. |
