# Supplementary material for: Bacterial Involvement in Oral Squamous Cell Carcinoma and Potentially Malignant Oral Disorders
Source: Oral Dis. 2025 Oct 9;32(4):992–1003. doi: 10.1111/odi.70115 (PMC13248574; doi:10.1111/odi.70115)
Supplement: Supplementary file 4 — Table S4: Alpha‐diversity metrics. Richness is the number of pure species found in the saliva samples from each group. [file ODI-32-992-s002.docx]

Table S4. Alpha-diversity metrics. Richness is the number of pure species found in the saliva samples from each group

| Richness (Observed features) | |  |  |  |  |  |
| --- | --- | --- | --- | --- | --- | --- |
|  |  | Minimum | Maximum | Median | SD | SE |
|  | Control | 15.00 | 95.00 | 72.5 | 14.97 | 2.12 |
|  | Lichen planus | 36.00 | 95.00 | 68.0 | 12.33 | 2.06 |
|  | Leukoplakia | 35.00 | 100.00 | 65.0 | 14.52 | 2.27 |
|  | Early OSCC | 48.00 | 92.00 | 69.0 | 11.36 | 1.73 |
|  | Advanced OSCC | 47.00 | 104.00 | 79.0 | 14.62 | 3.19 |
|  |  |  |  |  |  |  |
| Shannon entropy | |  |  |  |  |  |
|  |  | Minimum | Maximum | Median | SD | SE |
|  | Control | 3.64 | 5.84 | 5.18 | 0.43 | 0.06 |
|  | Lichen planus | 2.45 | 5.62 | 4.69 | 0.57 | 0.09 |
|  | Leukoplakia | 3.46 | 5.53 | 4.74 | 0.47 | 0.07 |
|  | Early OSCC | 3.56 | 5.52 | 4.76 | 0.41 | 0.06 |
|  | Advanced OSCC | 3.98 | 5.74 | 4.95 | 0.51 | 0.11 |
|  |  |  |  |  |  |  |
| Faith PD | |  |  |  |  |  |
|  |  | Minimum | Maximum | Median | SD | SE |
|  | Control | 7.09 | 13.85 | 11.12 | 1.27 | 0.18 |
|  | Lichen planus | 8.38 | 16.97 | 11.18 | 1.76 | 0.29 |
|  | Leukoplakia | 7.65 | 19.01 | 10.97 | 2.44 | 0.38 |
|  | Early OSCC | 8.29 | 17.47 | 11.30 | 1.73 | 0.26 |
|  | Advanced OSCC | 9.94 | 17.39 | 12.25 | 1.86 | 0.40 |
