# Supplementary material for: Bacterial Involvement in Oral Squamous Cell Carcinoma and Potentially Malignant Oral Disorders
Source: Oral Dis. 2025 Oct 9;32(4):992–1003. doi: 10.1111/odi.70115 (PMC13248574; doi:10.1111/odi.70115)
Supplement: Supplementary file 5 — Table S5: Beta diversity evaluated using PcoA. [file ODI-32-992-s006.docx]

Table S5. Beta diversity evaluated using PcoA

| Unweighted | |  |  |  |  |  |
| --- | --- | --- | --- | --- | --- | --- |
|  |  | PCOA1 Average | PCOA1 SE |  | PCOA2 Average | PCOA2 SE |
|  | Control | -0.03192 | 0.00458 |  | -0.00006 | 0.01168 |
|  | Lichen planus | -0.00190 | 0.02001 |  | 0.01623 | 0.01527 |
|  | Leukoplakia | 0.02648 | 0.02425 |  | 0.03177 | 0.01365 |
|  | Early OSCC | 0.00486 | 0.01654 |  | -0.01093 | 0.01331 |
|  | Advanced OSCC | 0.01761 | 0.02003 |  | -0.06732 | 0.01671 |
|  |  |  |  |  |  |  |
| Weighted | |  |  |  |  |  |
|  |  | PCOA1 Average | PCOA1 SE |  | PCOA2 Average | PCOA2 SE |
|  | Control | 0.01938 | 0.01900 |  | -0.02873 | 0.01399 |
|  | Lichen planus | 0.01086 | 0.02295 |  | 0.03648 | 0.01914 |
|  | Leukoplakia | 0.01816 | 0.02273 |  | 0.02536 | 0.01557 |
|  | Early OSCC | -0.03953 | 0.01644 |  | 0.01153 | 0.01512 |
|  | Advanced OSCC | -0.01927 | 0.01958 |  | -0.06726 | 0.02049 |
