# Supplementary material for: Bacterial Involvement in Oral Squamous Cell Carcinoma and Potentially Malignant Oral Disorders
Source: Oral Dis. 2025 Oct 9;32(4):992–1003. doi: 10.1111/odi.70115 (PMC13248574; doi:10.1111/odi.70115)
Supplement: Supplementary file 6 — Table S6: Relative abundance of Bacilli, Actinomycetia and Clostridia at the class level among five groups. [file ODI-32-992-s004.docx]

Table S6. Relative abundance of *Bacilli*, *Actinomycetia* and *Clostridia* at the class level among five groups

| Relative abundance of *Bacilli* | |  |  |  |  |  |
| --- | --- | --- | --- | --- | --- | --- |
|  |  | Minimum | Maximum | Average | SD | SE |
|  | Control | 15.18 | 62.34 | 35.27 | 10.14 | 1.43 |
|  | Lichen planus | 16.54 | 84.18 | 44.22 | 11.81 | 1.97 |
|  | Leukoplakia | 19.51 | 67.69 | 41.85 | 10.45 | 1.63 |
|  | Early OSCC | 16.97 | 67.63 | 44.60 | 11.24 | 1.71 |
|  | Advanced OSCC | 15.46 | 67.90 | 39.24 | 12.72 | 2.78 |
|  |  |  |  |  |  |  |
| Relative abundance of *Actinomycetia* | |  |  |  |  |  |
|  |  | Minimum | Maximum | Average | SD | SE |
|  | Control | 0.31 | 19.14 | 10.35 | 4.60 | 0.65 |
|  | Lichen planus | 2.22 | 35.66 | 11.01 | 6.51 | 1.08 |
|  | Leukoplakia | 3.41 | 30.32 | 11.91 | 6.80 | 1.06 |
|  | Early OSCC | 1.27 | 28.07 | 10.09 | 6.86 | 1.05 |
|  | Advanced OSCC | 0.45 | 21.81 | 5.53 | 5.71 | 1.25 |
|  |  |  |  |  |  |  |
|  | Relative abundance of *Clostridia* |  |  |  |  |  |
|  |  | Minimum | Maximum | Average | SD | SE |
|  | Control | 1.39 | 17.79 | 5.50 | 3.04 | 0.43 |
|  | Lichen planus | 0.05 | 10.98 | 4.70 | 2.79 | 0.46 |
|  | Leukoplakia | 0.21 | 8.88 | 3.94 | 2.02 | 0.36 |
|  | Early OSCC | 0.67 | 13.16 | 3.06 | 3.07 | 0.47 |
|  | Advanced OSCC | 1.31 | 30.67 | 9.04 | 7.77 | 1.70 |
|  |  |  |  |  |  |  |
